# Levothyroxine Therapy in Thyrodectomized Patients

**DOI:** 10.3389/fendo.2020.626268

**Published:** 2021-01-29

**Authors:** Paolo Miccoli, Gabriele Materazzi, Leonardo Rossi

**Affiliations:** Department of Surgical, Medical, Molecular and Critical Pathology, University of Pisa, Pisa, Italy

**Keywords:** thyroid, levothyroxine, thyroidectomy, liquid levothyroxine, levothyroxine dose

## Abstract

Administration of the optimal dose of levothyroxine (LT4) is crucial to restore euthyroidism after total thyroidectomy. An insufficient or excessive dosage may result in hypothyroidism or thyrotoxicosis, either one associated with a number of symptoms/complications. Most literature regarding the LT4 dosage deals with the treatment of primary hypothyroidism, whereas a limited number of studies handle the issue of thyroxin replacement after total thyroidectomy. A literature review was performed focusing on all papers dealing with this topic within the last 15 years. Papers that reported a scheme to calculate the proper LT4 dose were collected and compared to set up a review exploring limits and drawbacks of LT4 replacement therapy in the wide population of patients who had undergone thyroidectomy. Most of the methods for monitoring and adjusting thyroid hormone replacement after thyroidectomy for benign disease use LT4 at an empirical dose of approximately 1.6 μg/kg, with subsequent changes according to thyroid function test results and assessments of the patient’s symptoms. Approximately 75% of patients require a dose adjustment, suggesting that factors other than body weight play a role in the determination of the proper LT4 dose. Hence, several schemes are reported in the literature for the proper initial dose of LT4. An inadequate level of thyroid hormone levels in these patients can be due to several factors. The most common ones that lead to the necessity of LT4 dose adjustments include lack of compliance, changes in LT4 formulation, dosage errors, increased serum levels of T4-binding globulin, body mass changes, and dietary habits. Moreover, concomitant ingestion of calcium supplements, ferrous sulfate, proton-pump inhibitors, bile acid sequestrants, and sucralfate might influence LT4 absorption and/or metabolism. Furthermore, some gastrointestinal conditions and their treatments can contribute to suboptimal LT4 performance by altering gastric acidity and thereby reducing its bioavailability, particularly in the solid form. Beyond the classic tablet form, new formulations of LT4, such as a soft gel capsule and an oral solution, recently became available. The liquid formulation is supposed to overcome the food and beverages interference with absorption of LT4 tablets.

## Introduction

There is a wide consensus among different authors that a relevant percentage of patients taking levothyroxine (LT4) for hypothyroidism induced by different causes show a non-perfect compliance with therapy that necessitates several changes and adjustments during their treatment (1, 2). Inadequate dosage may result in hypothyroidism or hyperthyroidism, both involving serious sequelae at several different levels. Overtreatment causes hyperthyroidism and associated cardiac symptoms, weight loss, insomnia, and heat intolerance (3). On the other hand, overt and subclinical hypothyroidism have both been associated with unfavorable changes in several metabolic parameters, including lipid profile and glucose control, as well as with higher blood pressure and insulin resistance, all conditions that may amplify the cardiovascular disease risk in type 2 diabetes. Similar associations have also been reported for thyroid-stimulating hormone (TSH) levels in the upper part of the normal reference range (4, 5).

This review examined the literature involving hypothyroid patients who underwent a total or near-total thyroidectomy to understand the problems they generally encounter with their replacement therapy. For this purpose, the literature review focused on all papers dealing with this topic during the last 15 years. Only eight of these studies also contained a proposal to work out a scheme for the prediction of the LT4 requirement after thyroidectomy.

Common factors that can lead to LT4 dose adjustments include lack of compliance, changes in the LT4 formulation, dosage errors, increased serum levels of T4-binding globulin, body mass changes, and dietary habits (1, 6). Concomitant ingestion of calcium supplements, ferrous sulfate, proton-pump inhibitors, bile acid sequestrants, and sucralfate can also influence LT4 absorption and/or metabolism (6, 7).

LT4 is a medication with a narrow therapeutic index, and its absorption is dependent on gastric pH (1, 6). Indeed, acid production is reducted in patients with chronic gastritis or gastric atrophy, in those treated with proton-pump inhibitors, and in patients with *Helicobacter pylori* infection: all of these conditions have been related to increased thyroxine requirement (6).

Some gastrointestinal conditions and their treatments can contribute to suboptimal LT4 performance by altering gastric acidity and thereby reducing the bioavailability of LT4. Defects in absorption of thyroid hormones are reported in patients with previous gut surgery, celiac disease, lactose intolerance, autoimmune gastritis, or *Helicobacter pylori* infection (6, 8, 9).

Most of the debate in the past was focused on LT4 dosage and possibly on the timing of the administration. More recently, however, increasing attention has also been paid to the formulation of LT4. The liquid form in two presentations—a soft gel capsule and as an oral solution—recently became available for LT4 replacement therapy (10). Notably, a population-based study of 55,000 LT4 users reported a significant reduction in the number of TSH measurements after switching from the tablet to the liquid form, particularly in patients using drugs that potentially interfere with LT4 absorption (11). These papers addressing the liquid form report some promising results also in patients who have undergone thyroidectomy (12, 13).

Another important issue is the possible necessity of a suppressive thyroid hormone treatment in those patients who have undergone a total thyroidectomy for a differentiated thyroid cancer (DTC). This might imply a long-term treatment with LT4 to the extent that in these patients it is even more important to individualize the therapy to balance accurately its supposed benefits against the potential risk of adverse effects during follow-up. A proper selection then becomes of paramount importance (8). American Thyroid Association (ATA) guidelines help in determining the class of risk in patients, mainly represented by the presence of distant metastases, and the response to the initial treatment. On the other hand, advanced age, risk factors, and underlying comorbidities might play an important role against TSH suppression, and the latter should be avoided (14, 15). For these reasons and because of the low risk of recurrence and death in DTC, ATA recommends a graded algorithm in which the potential benefits of this therapy are carefully weighed against its cardiovascular and skeletal risks (14, 15).

## Literature Review

A literature review focused on papers reporting LT4 treatment in patients after a total or near total thyroidectomy. Data were collected and scrutinized from the PubMed database using combinations of the following search terms: levothyroxine, dosing, thyroidectomy, differentiated thyroid cancer, and liquid levothyroxine. Additional studies were selected through reference lists of articles known to be relevant. We only considered articles that were published in the last 15 years and investigated LT4 treatment in adults (aged ≥18 years) after total, near-total, or completion thyroidectomy. Only articles written in English were included.

### LT4 Dosing Schemes After Total Thyroidectomy

Most of literature regarding the LT4 dosage deals with the treatment of primary hypothyroidism, whereas only few studies handle the issue of thyroxine replacement after total thyroidectomy (16). As reported by Del Duca et al. (17) in a longitudinal study including 23 goitrous patients treated with LT4, the therapeutic dose of T4 after total thyroidectomy must be increased by one-third compared with the presurgical one. This additional amount of T4 may be the substrate for the peripheral deiodinase network to compensate the absence of T3 production from the gland (17).

Most of the methods for monitoring and adjusting thyroid hormone replacement after thyroidectomy for benign disease use LT4 at an empirical dose of approximately 1.6 μg/kg, with subsequent changes based on thyroid function test results and assessments of the patient’s symptoms (2). Approximately 75% of patients require dose adjustments, suggesting that body weight is not the only factor involved in the determination of the proper LT4 dose (3). On the basis of our literature review, we singled out eight schemes proposed to calculate the proper dose of LT4 after thyroidectomy (**Table 1**). These schemes take into consideration a combination of the following parameters: body weight, age, body surface area (BSA), iron supplementation, mineral supplementation, preoperative TSH concentration, sex, or body mass index (BMI). The heterogeneity of these schemes implies the contribution of several parameters to the LT4 dose calculation and raises the need for an accurate, simple, and widely shared formula.

**Table 1:** Main proposed schemes for the prediction of LT4 requirement after total thyroidectomy.

Study	Type of study	Number of patients	Formula/nomogram
Olobuwale et al. (16)	Prospective	27	LT4 dose (μg/day) = - 100 if weight <53 kg - 125 if 53 ≤ weight ≤ 86 kg - 150 if 86 < weight ≤ 108 kg - 175 if weight >108 kg
Mistry et al. (2)	Prospective	100	LT4 dose (μg/day) = (0.943 × weight) + (–1.165 × age) + 125.8 (Simplified to = weight – age + 125)
Ojomo et al. (7)	Retrospective	122	LT4 dose (μg/day) = (–0.018 × BMI + 2.13) × weight
Jin et al. (18)	Retrospective	400	LT4 dose (μg/day) = 1.5 × weight
Di Donna et al. (19)	Prospective	31	LT4 dose (μg/day) = BMI ≤23 23–28 >28 Age ≤40 1.8 1.7 1.6 >40–55 1.7 1.6 1.5 >55 1.6. 1.5. 1.4
Elfenbein et al. (20)	Prospective	180	LT4 dose (μg/day) = Male Female BMI (kg/m^2^) ≤ 21 2.1 1.8 22–26 1.9 1.7 27-32 1.7 1.6 33-40 1.5 1.4 >40 1.3 1.2
Zaborek et al. (21)	Retrospective	598	LT4 dose (μg/day) = e^x^ x = 2.02 + 0.01 (weight) – 0.0037 (age) – 0.098 (sex) – 0.01 (BMI) + 0.007 (preoperative TSH) + 0.108 (iron supplementation) – 0.014 (mineral supplementation) Note: patient sex: 1 for female, 0 for male; iron supplementation: 1 for supplementation, otherwise 0; mineral supplementation: 1 for supplementation, otherwise 0.
Al Dahiri et al. (22)	Retrospective	234	LT4 dose (μg/day) = - If BSA > 1.79 m^2^ 1.4 x weight - If BSA ≤ 1.79 m^2^ 1.7 x weight

In particular, Olobuwale et al. (16) and Jin et al. (18) developed two different weight-based schemes to calculate the proper LT4 dose in patients who underwent thyroidectomy. Nevertheless, when retrospectively applied by other authors, the rate of patients being reported to be euthyroid at the first postsurgery follow-up was amendable, ranging between 23 and 53.2% (19, 21). Furthermore, Jin et al. (18) included patients who had undergone total thyroidectomy or lobectomy, making their data not homogeneous.

Successively, other schemes were proposed. Mistry et al. (2) introduced age as a factor to be considered along with body weight to calculate the proper LT4 dose. They compared their formula to the empirical dose of 100 μg and to the only weight-based dose calculation (1.6 μg × kg), finding that 72, 59, and 40% of patients, respectively, achieved the target within 25 μg of their proper dose. Even this formula, when applied retrospectively, did not lead to better results.

A remarkable improvement was obtained when schemes using BMI were introduced. First, Ojomo et al. (7) proposed a formula that takes into consideration both BMI and actual body weight, with optimal results. This formula was applied retrospectively and compared with the other existing schemes, both by Di Donna et al. and Zaborek et al. and resulted as the most accurate method (19). Afterward, Di Donna et al. and Elfenbein et al. produced schemes that take into account age and sex categories, respectively, along with BMI (19, 20) Di Donna et al applied their scheme prospectively to a cohort of 31 patients, achieving euthyroid status at the first follow-up in 68% (19). On the other hand, when the formula proposed by Elfenbein et al. was retrospectively applied to a cohort of 180 patients, it predicted the eventual euthyroid dose of LT4 to within 20 μg for 61% of patients (20).

An important step forward was made by Zaborek et al. who developed a Poisson regression formula that takes into account seven factors: four of them were found in previous proposed schemes (actual body weight, BMI, age, sex), whereas the remaining three additional factors were preoperative TSH, vitamin-mineral supplementation, and iron supplementation (21). In this study of a retrospective cohort of 598 patients, their scheme outperformed the other previously proposed schemes, predicting 64.8% of the corrected doses at the first follow-up. To promote the use of Poisson regression to estimate the proper LT4 dose, the authors developed an easy-to-use web application that allows the physicians to insert the patient’s parameters and automatically calculate the LT4 dose.

Finally, in 2019, Al Dhahiri et al. (22) performed a study to identify factors that would predict the LT4 dose after thyroidectomy. Their analysis showed BSA as an independent predictor of the LT4 dose. The authors developed two different and very complex formulas (one polynomial and one linear) for predicting the LT4 dose after thyroidectomy, with a rate of correct estimation of 65.8 and 51.3%, respectively. They also created a model that takes into consideration only BSA as a unique parameter, competent to predict 64.5% of the corrected dose. Nevertheless, due to the complexity of calculating the dose, finding a practical and clinically relevant prediction model is yet of limited efficiency.

### Differentiated Thyroid Cancer

Thyroid hormone treatment plays a central role in the postoperative management of DTC. Nevertheless, long-term treatment with LT4 should be individualized and weighed against the potential risk of adverse effects, and patients who require suppressive therapy should be appropriately selected (8). Thus, before LT4 therapy is administered, it is of paramount importance to consider, along with the risk of persistent or recurrent disease and the response to the initial treatment, whether poor general health status and comorbidities (in particular, bone and cardiac diseases) contraindicate TSH suppression (14). Furthermore, Jin et al. reported that TSH-suppression therapy after thyroidectomy due to DTC could lead to depression, short-term memory and attention impairment, and word selection anomia (18).

Given the evidence that aggressive TSH-suppressive therapy led to little or even no benefit in almost all patients who underwent total thyroidectomy for DTC, ATA guidelines recommended a scheme in which the potential advantages of this therapy are carefully balanced against its cardiovascular and skeletal collateral effects (7, 8). Especially in elderly patients, who have an increased risk of osteoporosis and atrial fibrillation, the TSH target should be carefully selected (23).

According to the most recent Italian Consensus on Diagnosis and Treatment of DTC, after the initial therapy, patients included in the ATA high-risk category should be maintained at TSH suppression below 0.1 mU/L, unless contraindicated by comorbidities or advanced age (shift the target to 0.1–0.5 mU/L). Patients included in the ATA intermediate-risk category should be maintained at TSH levels between 0.1 and 0.5 mU/L. Finally, patients included in the ATA low-risk category, with undetectable thyroglobulin, should maintain a TSH level between 0.5 and 2 mU/L, whereas the level decreases to 0.1–0.5 mU/L if the thyroglobulin is low. In case of comorbidities or advanced age, the low- and intermediate-risk categories should both maintain TSH levels between 0.5 and 2 mU/L (24).

Concerning the degree of suppression during follow-up, regardless the initial ATA class risk classification, patients with excellent response to therapy should maintain a TSH level between 0.5 and 2 mU/L, regardless of comorbidities and age. Furthermore, patients with biochemical incomplete or indeterminate response should maintain a TSH level between 0.1 and 0.5 mU/L, which increases to a level between 0.5 and 2 mU/L in case of comorbidities or advanced age. Finally, patients with a structural incomplete response should maintain a TSH level lower than 0.1 mU/L, unless there are comorbidities or advanced age, which would shift the target to 0.1–0.5 mU/L (24).

Further, Carhill et al. (25) in 2015 analyzed a registry of 4,941 patients with DTC who had undergone a total thyroidectomy or near-total thyroidectomy with a median follow-up of 6 years. They reported that moderate thyroid hormone suppressive therapy, with TSH maintained in subnormal to normal levels, was associated with an improvement of overall survival and disease-free survival across all stages of DTC and that aggressive thyroid hormone suppressive therapy (TSH maintained undetectable-subnormal) showed no additional survival benefit (25).

In a study of 148 consecutive patients who underwent total thyroidectomy for DTC, Ito et al. investigated the relationship between symptoms and serum TSH and FT3 levels (26). Symptoms reflecting thyroid function were documented and compared preoperatively and postoperatively after 12 months of LT4 therapy. The authors found that in patients with strongly suppressed TSH levels, significant changes in symptoms with a tendency toward thyrotoxicosis were reported. However, patients with normal TSH levels experienced changes in symptoms with a tendency toward hypothyroidism. Lastly, in patients with mildly suppressed TSH levels and FT3 levels superimposable to the preoperative values, all symptoms remained equivalent to their preoperative levels. On the basis of their study, they claimed that patients with mildly suppressed TSH levels were closer to the euthyroid status and suggested that these finding were directly applicable to the management of patients who underwent total thyroidectomy for DTC or benign thyroid disease (26).

### Liquid and Soft Gel Formulations

Beyond the classic tablet, new formulations of thyroxine can now be prescribed as a soft gel capsule and oral solution, which have been shown to overcome the food and beverages interference with absorption of LT4 tablets. In addition, the liquid formulation was of particular interest in case of malabsorption resulting from atrophic gastritis, proton-pump inhibitors, or after bariatric surgery. Malabsorption induced by lactose intolerance or drug interference can also be avoided (12). In particular, Benvenga et al. (27) addressed this topic in a study of 19 hypothyroid patients with tablet LT4 malabsorption caused by calcium and/or iron supplements and who were switched to liquid LT4 at the same dose. The authors reported that the TSH level was lower with the liquid LT4 compared with tablet LT4 form, concluding that liquid LT4 is resistant to the sequestration by calcium or iron. Moreover, the high rate of TSH normalization at the first check should avoid frequent adjustments in LT4 doses, with consequent financial savings.

The liquid LT4 also seems to be more active than tablets in the control of TSH even in hypothyroid patients without malabsorption, drug interference, or gastric disorders, leading to the hypothesis that absorption of liquid LT4 is also higher in this cohort (12).

Our literature review resulted in eight papers dealing with the administration of a liquid or soft gel capsule formulation in patients who underwent total thyroidectomy. These studies are summarized in **Table 2**.

**Table 2:** Prospective studies dealing with liquid or soft gel formulation of LT4 administered post total thyroidectomy.

Study	Number of patients	Treatment groups	Main findings
Pirola et al. (28)	20	Liquid *vs*. tablet LT4 in patients with enteral feeding tube	- Liquid LT4 can be administered immediately without the need for an empty stomach. This formulation is more easily managed by nurses
Giusti et al. (29)	59	Liquid *vs*. tablet LT4 in patients with DTC	- Subjective complaints were significantly lower on the liquid (28%) than on the tablet (43%) L-T4 formulation - 73% requested to remain on the liquid formulation
Cappelli et al. (30)	60	Liquid *vs*. soft gel LT4 during breakfast	- Both formulations can be administered during breakfast - FT3 (2.5 *vs*. 2.7 pg/mL) and FT4 (9.9 *vs*. 10.6 pg/mL) levels during treatment with the soft gel capsule were significantly lower than those at enrollment with the liquid LT4 formulation
Lombardi et al. (31)	166	Liquid *vs*. tablet LT4	- Liquid LT4 could be more effective in improving mood states and self-perception of well-being
Cappelli et al. (13)	102	Liquid *vs*. tablet LT4 in patients with DTC	- 14.2% of patients on tablets and 3.9% of patients on liquid formulation showed TSH levels > 0.5 mIU/L
Di Donna et al. (32)	103	Soft gel *vs* tablet LT4	- LT4 requirement is not significantly different, but TSH is significantly lower with the use of the soft gel capsule, 1.3 ± 0.9 *vs*. 1.8 ± 1.2, respectively
Fallahi et al. (12)	105	Liquid *vs*. tablet LT4 in patients with DTC	- The prevalence of patients in the hypothyroid range was significantly higher in the LT4 tablet group (13.5%) *vs*. the liquid LT4 group (1.8%)
Peirce et al. (33)	3	Liquid *vs*. tablet LT4 in patients with severe hypothyroidism after total thyroidectomy	All patients (100%) were restored to euthyroidism after switching from LT4 tablets to LT4 liquid form

Fallahi et al. (12) in 2018 conducted a prospective study of 105 patients who underwent total thyroidectomy for thyroid cancer, 52 of whom were treated with the liquid LT4 formulation and 53 with LT4 tablets at the same dosage (1.5 µg/kg/day). TSH levels were significantly lower in patients treated with liquid LT4, suggesting a higher absorption with this formulation (12). In particular, the rate of patients in the hypothyroid range was significantly higher in the solid form LT4 group (13.5%) compared with the liquid LT4 group (1.8%). The authors underlined the effectiveness of liquid LT4 over the tablet formulation to achieve the proper TSH levels in patients treated with total thyroidectomy for thyroid cancer.

Similarly, Cappelli et al. (13) performed a prospective randomized study of 102 patients who underwent total thyroidectomy and radioactive iodine therapy for DTC and were classified as low risk according to the 2009 ATA guidelines. The use of tablets, compared with treatment with the LT4 liquid formulation, resulted in a higher number of DTC patients with TSH values out of range for the ATA risk score (15.7 *vs*. 3.9%) during 24 months of follow-up. Moreover, no body weight changes were observed among the whole population enrolled.

A study by Peirce et al. (33) in 2018 of patients who underwent total thyroidectomy with severe hypothyroidism reported that euthyroidism was progressively restored after sublingual administration of liquid LT4, making this formulation a valid alternative method for acute treatment of severe hypothyroidism.

Further, Giusti et al. (29) conducted a study of 59 patients with cured DTC who were switched from the tablet to the liquid formulation of LT4 and found no change in TSH, thyroid hormones, and thyroglobulin levels during the study. Although significantly more patients found the tablet form more agreeable, subjective symptoms had decreased significantly at the end of the study, and 73% of patients requested to remain on the liquid formulation (29). The authors concluded that liquid LT4 could be considered as a valid alternative formulation in patients after thyroidectomy for DTC and that the initial dislike by the patients was overcome by a significant decrease in subjective symptoms.

Lombardi et al. (31) performed a prospective randomized study in 2016 of 155 patients who had undergone total thyroidectomy and evaluated the patients’ mood state and their self-perception of mental well-being during a follow-up period of 2 months. Their study found that the liquid formulation resulted in a significantly greater efficacy in ameliorating these parameters.

Other notable aspects were evaluated by Pirola et al. (28). They recruited 20 patients who underwent laryngectomy along with thyroidectomy and who were randomized in two groups, one was treated with LT4 tablets, and one was treated with LT4 liquid formulation. The authors concluded that the liquid form of LT4 can be administered directly through the nasogastric tube without the need for an empty stomach and is more easily managed by a nurse.

In addition to the liquid LT4, another new formulation of LT4 is represented by the soft gel capsule. In 2014, Di Donna et al. (32) studied 103 patients who had undergone total thyroidectomy for benign diseases. Once a stable normal TSH value was achieved using the tablet formulation, the patients were switched to treatment with the soft gel capsule at the same previous dose. The LT4 dose required for achieving normal TSH values did not differ between tablets and the soft gel capsule formulation. A statistically significant decrease of approximately 28% in the mean TSH level was documented with the use of the soft gel capsule formulation, which may represent an important advantage considering the possible association between elevated FT4 levels, even in euthyroid patients, and cardiovascular disease.

In a recent study, soft gel capsule and liquid formulations given during breakfast were compared for their effects on thyroid hormone profile. Although both formulations can be taken during breakfast, the liquid one should be preferred for patients in whom even small changes of thyroid hormones levels must be avoided (30).

## Conclusion

Despite a remarkable commitment by researchers to find a therapeutic scheme able to predict the exact dose of LT4 to be given to patients after a total thyroidectomy, the attempt to reach the precise dosage failed to reach the target in the totality of the cases. It is reasonable to conclude that because most of the schemes show a considerable complexity but do not offer significant advantages in the percentage of patients reaching the expected results, the search for a fully predictive model seems to be an exercise of futility. Nonetheless, these schemes are of great utility to start the replacement therapy approaching the best dosage, but keeping in mind that changes during follow-up can be necessary according to the TSH values that are to be reached in every case.

Finally, data from the review seem to demonstrate that a significant role will be played by the liquid formulation of LT4, mainly for two reasons: first, because its absorption is easier and quicker than the tablet format, and second, it facilitates a more rapid control of TSH in patients with any kind of malabsorption and probably also in patients without this problem.

## Author Contributions

LR wrote the manuscript with the support of PM and collected data for the literature review. Moreover, LR reviewed the final version of the manuscript. All authors contributed to the article and approved the submitted version.

## Conflict of Interest

The authors declare that the research was conducted in the absence of any commercial or financial relationships that could be construed as a potential conflict of interest.
